# Author Correction: TDP-43 loss and ALS-risk SNPs drive mis-splicing and depletion of UNC13A

**DOI:** 10.1038/s41586-024-07577-9

**Published:** 2024-06-18

**Authors:** Anna-Leigh Brown, Oscar G. Wilkins, Matthew J. Keuss, Sarah E. Kargbo-Hill, Matteo Zanovello, Weaverly Colleen Lee, Alexander Bampton, Flora C. Y. Lee, Laura Masino, Yue A. Qi, Sam Bryce-Smith, Ariana Gatt, Martina Hallegger, Delphine Fagegaltier, Hemali Phatnani, NYGC ALS Consortium, Hemali Phatnani, Justin Kwan, Dhruv Sareen, James R. Broach, Zachary Simmons, Ximena Arcila-Londono, Edward B. Lee, Vivianna M. Van Deerlin, Neil A. Shneider, Ernest Fraenkel, Lyle W. Ostrow, Frank Baas, Noah Zaitlen, James D. Berry, Andrea Malaspina, Pietro Fratta, Gregory A. Cox, Leslie M. Thompson, Steve Finkbeiner, Efthimios Dardiotis, Timothy M. Miller, Siddharthan Chandran, Suvankar Pal, Eran Hornstein, Daniel J. MacGowan, Terry Heiman-Patterson, Molly G. Hammell, Nikolaos. A. Patsopoulos, Oleg Butovsky, Joshua Dubnau, Avindra Nath, Robert Bowser, Matthew Harms, Eleonora Aronica, Mary Poss, Jennifer Phillips-Cremins, John Crary, Nazem Atassi, Dale J. Lange, Darius J. Adams, Leonidas Stefanis, Marc Gotkine, Robert H. Baloh, Suma Babu, Towfique Raj, Sabrina Paganoni, Ophir Shalem, Colin Smith, Bin Zhang, Brent Harris, Iris Broce, Vivian Drory, John Ravits, Corey McMillan, Vilas Menon, Lani Wu, Steven Altschuler, Yossef Lerner, Rita Sattler, Kendall Van Keuren-Jensen, Orit Rozenblatt-Rosen, Kerstin Lindblad-Toh, Katharine Nicholson, Peter Gregersen, Jeong-Ho Lee, Sulev Koks, Stephen Muljo, Jia Newcombe, Emil K. Gustavsson, Sahba Seddighi, Joel F. Reyes, Steven L. Coon, Daniel Ramos, Giampietro Schiavo, Elizabeth M. C. Fisher, Towfique Raj, Maria Secrier, Tammaryn Lashley, Jernej Ule, Emanuele Buratti, Jack Humphrey, Michael E. Ward, Pietro Fratta

**Affiliations:** 1 UCL Queen Square Motor Neuron Disease Centre, Department of Neuromuscular Diseases, UCL Queen Square Institute of Neurology, UCL, London, UK; 2 The Francis Crick Institute, London, UK; 3 National Institute of Neurological Disorders and Stroke, NIH, Bethesda, MD USA; 4 Queen Square Brain Bank, UCL Queen Square Institute of Neurology, University College London, London, UK; 5 Department of Neurodegenerative Disease, UCL Queen Square Institute of Neurology, University College London, London, UK; 6 Center for Alzheimer’s and Related Dementias, National Institutes of Health, Bethesda, MD USA; 7 Center for Genomics of Neurodegenerative Disease, New York Genome Center (NYGC), New York, NY USA; 8 NeuroResource, Department of Neuroinflammation, UCL Queen Square Institute of Neurology, London, UK; 9 Great Ormond Street Institute of Child Health, Genetics and Genomic Medicine, University College London, London, UK; 10 Medical Scientist Training Program, Johns Hopkins University School of Medicine, Baltimore, MD USA; 11 Molecular Genomics Core, Eunice Kennedy Shriver National Institute of Child Health and Human Development, NIH, Bethesda, MD USA; 12 UK Dementia Research Institute, University College London, London, UK; 13 Nash Family Department of Neuroscience and Friedman Brain Institute, Icahn School of Medicine at Mount Sinai, New York, NY USA; 14 Ronald M. Loeb Center for Alzheimer’s Disease, Icahn School of Medicine at Mount Sinai, New York, NY USA; 15 Department of Genetics and Genomic Sciences and Icahn Institute for Data Science and Genomic Technology, Icahn School of Medicine at Mount Sinai, New York, NY USA; 16 Estelle and Daniel Maggin Department of Neurology, Icahn School of Medicine at Mount Sinai, New York, NY USA; 17 Department of Genetics, Evolution and Environment, UCL Genetics Institute, University College London, London, UK; 18 Department of Molecular Biology and Nanobiotechnology, National Institute of Chemistry, Ljubljana, Slovenia; 19 Molecular Pathology Lab, International Centre for Genetic Engineering and Biotechnology (ICGEB), Trieste, Italy; 20 Department of Neurology, Lewis Katz School of Medicine, Temple University, Philadelphia, PA USA; 21 Cedars-Sinai Department of Biomedical Sciences, Board of Governors Regenerative Medicine Institute and Brain Program, Cedars-Sinai Medical Center, University of California, Los Angeles, CA USA; 22 Department of Medicine, University of California, Los Angeles, CA USA; 23 Department of Biochemistry and Molecular Biology, Penn State Institute for Personalized Medicine, The Pennsylvania State University, Hershey, PA USA; 24 Department of Neurology, The Pennsylvania State University, Hershey, PA USA; 25 Department of Neurology, Henry Ford Health System, Detroit, MI USA; 26 Department of Pathology and Laboratory Medicine, Perelman School of Medicine, University of Pennsylvania, Philadelphia, PA USA; 27 Department of Neurology, Center for Motor Neuron Biology and Disease, Institute for Genomic Medicine, Columbia University, New York, NY USA; 28 Department of Biological Engineering, Massachusetts Institute of Technology, Cambridge, MA USA; 29 Department of Neurology, Johns Hopkins School of Medicine, Baltimore, MD USA; 30 Department of Neurogenetics, Academic Medical Centre, Amsterdam, The Netherlands; 31 Leiden University Medical Center, Leiden, The Netherlands; 32 Department of Medicine, Lung Biology Center, University of California, San Francisco, CA USA; 33 ALS Multidisciplinary Clinic, Neuromuscular Division, Department of Neurology, Harvard Medical School, Boston, MA USA; 34 Neurological Clinical Research Institute, Massachusetts General Hospital, Boston, MA USA; 35 Centre for Neuroscience and Trauma, Blizard Institute, Barts, Queen Mary University of London, London, UK; 36 The London School of Medicine and Dentistry, Queen Mary University of London, London, UK; 37 Department of Neurology, Basildon University Hospital, Basildon, UK; 38 The Jackson Laboratory, Bar Harbor, ME USA; 39 Department of Psychiatry and Human Behavior, Department of Biological Chemistry, School of Medicine, University of California, Irvine, CA USA; 40 Department of Neurobiology and Behavior, School of Biological Sciences, University of California, Irvine, CA USA; 41 Taube/Koret Center for Neurodegenerative Disease Research, Roddenberry Center for Stem Cell Biology and Medicine, Gladstone Institute, San Francisco, CA USA; 42 Department of Neurology and Sensory Organs, University of Thessaly, Thessaly, Greece; 43 Department of Neurology, Washington University in St Louis, St Louis, MO USA; 44 Centre for Clinical Brain Sciences, Anne Rowling Regenerative Neurology Clinic, Euan MacDonald Centre for Motor Neurone Disease Research, University of Edinburgh, Edinburgh, UK; 45 Department of Molecular Genetics, Weizmann Institute of Science, Rehovot, Israel; 46 Department of Neurology, Icahn School of Medicine at Mount Sinai, New York, NY USA; 47 Center for Neurodegenerative Disorders, Department of Neurology, the Lewis Katz School of Medicine, Temple University, Philadelphia, PA USA; 48 Cold Spring Harbor Laboratory, Cold Spring Harbor, NY USA; 49 Ann Romney Center for Neurologic Diseases, Brigham and Women’s Hospital, Harvard Medical School, Boston, MA USA; 50 Department of Anesthesiology, Stony Brook University, Stony Brook, NY USA; 51 Section of Infections of the Nervous System, National Institute of Neurological Disorders and Stroke, NIH, Bethesda, MD USA; 52 Department of Neurology, Barrow Neurological Institute, St Joseph’s Hospital, Phoenix, AZ USA; 53 Medical Center, Department of Neurobiology, Barrow Neurological Institute, St Joseph’s Hospital and Medical Center, Phoenix, AZ USA; 54 Department of Neurology, Division of Neuromuscular Medicine, Columbia University, New York, NY USA; 55 Department of Neuropathology, Academic Medical Center, University of Amsterdam, Amsterdam, The Netherlands; 56 Department of Biology and Veterinary and Biomedical Sciences, The Pennsylvania State University, University Park, PA USA; 57 New York Stem Cell Foundation, Department of Bioengineering, School of Engineering and Applied Sciences, University of Pennsylvania, Philadelphia, PA USA; 58 Department of Pathology, Fishberg Department of Neuroscience, Icahn School of Medicine at Mount Sinai, New York, NY USA; 59 Department of Neurology, Harvard Medical School, Neurological Clinical Research Institute, Massachusetts General Hospital, Boston, MA USA; 60 Department of Neurology, Hospital for Special Surgery, New York, NY USA; 61 Weill Cornell Medical Center, New York, NY USA; 62 Medical Genetics, Atlantic Health System, Morristown Medical Center, Morristown, NJ USA; 63 Overlook Medical Center, Summit, NJ USA; 64 Center of Clinical Research, Experimental Surgery and Translational Research, Biomedical Research Foundation of the Academy of Athens (BRFAA), Athens, Greece; 65 1st Department of Neurology, Eginition Hospital, Medical School, National and Kapodistrian University of Athens, Athens, Greece; 66 Neuromuscular/EMG service and ALS/Motor Neuron Disease Clinic, Hebrew University-Hadassah Medical Center, Jerusalem, Israel; 67 Board of Governors Regenerative Medicine Institute, Los Angeles, CA USA; 68 Department of Neurology, Cedars-Sinai Medical Center, Los Angeles, CA USA; 69 Harvard Medical School, Department of Physical Medicine and Rehabilitation, Spaulding Rehabilitation Hospital, Boston, MA USA; 70 Center for Cellular and Molecular Therapeutics, Children’s Hospital of Philadelphia, Philadelphia, PA USA; 71 Department of Genetics, Perelman School of Medicine, University of Pennsylvania, Philadelphia, PA USA; 72 Centre for Clinical Brain Sciences, University of Edinburgh, Edinburgh, UK; 73 Euan MacDonald Centre for Motor Neurone Disease Research, University of Edinburgh, Edinburgh, UK; 74 Department of Genetics and Genomic Sciences, Icahn Institute of Data Science and Genomic Technology, Icahn School of Medicine at Mount Sinai, New York, NY USA; 75 Department of Neuropathology, Georgetown Brain Bank, Georgetown Lombardi Comprehensive Cancer Center, Georgetown University Medical Center, Washington, DC USA; 76 Neuroradiology Section, Department of Radiology and Biomedical Imaging, University of California, San Francisco, San Francisco, CA USA; 77 Neuromuscular Diseases Unit, Department of Neurology, Tel Aviv Sourasky Medical Center, Sackler Faculty of Medicine, Tel-Aviv University, Tel-Aviv, Israel; 78 Department of Neuroscience, University of California San Diego, La Jolla, CA USA; 79 Department of Neurology, University of Pennsylvania Perelman School of Medicine, Philadelphia, PA USA; 80 Department of Neurology, Columbia University Medical Center, New York, NY USA; 81 Department of Pharmaceutical Chemistry, University of California San Francisco, San Francisco, CA USA; 82 Hadassah Hebrew University, Jerusalem, Israel; 83 Department of Translational Neuroscience, Barrow Neurological Institute, Phoenix, AZ USA; 84 The Translational Genomics Research Institute (TGen), Phoenix, AZ USA; 85 Broad Institute, Cambridge, MA USA; 86 Massachusetts General Hospital, Boston, MA USA; 87 Institute of Molecular Medicine, Feinstein Institutes for Medical Research, Northwell Health, Manhasset, NY USA; 88 Korea Advanced Institute of Science and Technology (KAIST), Daejeon, South Korea; 89 Perron Institute for Neurological and Translational Science, Nedlands, Western Australia Australia; 90 Integrative Immunobiology Section, National Institute of Allergy and Infectious Disease, NIH, Bethesda, MD USA

**Keywords:** Amyotrophic lateral sclerosis, Neurodegeneration

Correction to: *Nature* 10.1038/s41586-022-04436-3 Published online 23 February 2022

The version of the article initially published referred to one, cell line as SK-N-DZ. This line has since been conclusively identified as SK-N-BE(2). The HTML and PDF versions of the article have been updated to reflect this.

