# Supplementary material for: TDP-43 loss and ALS-risk SNPs drive mis-splicing and depletion of UNC13A
Source: Nature. 2022 Feb 23;603(7899):131–7. doi: 10.1038/s41586-022-04436-3 (PMC8891020; doi:10.1038/s41586-022-04436-3)

nature portfolio

https://doi.org/10.1038/s41586-022-04436-3

Supplementary information

TDP-43 loss and ALS-risk SNPs drive mis-splicing and depletion of UNC13A

In the format provided by the authors and unedited

nature portfolio

https://doi.org/10.1038/s41586-022-04436-3

Supplementary information

TDP-43 loss and ALS-risk SNPs drive mis-splicing and depletion of UNC13A

In the format provided by the
authors and unedited

Supplemental Figure 1. Uncropped immunoblots from Extended Data Fig. 3F. Red dashed boxes indicate regions shown in the figure. UNC13B, Tubulin, and TDP-43 are from the same membrane. UNC13A is blotted on a separate membrane.

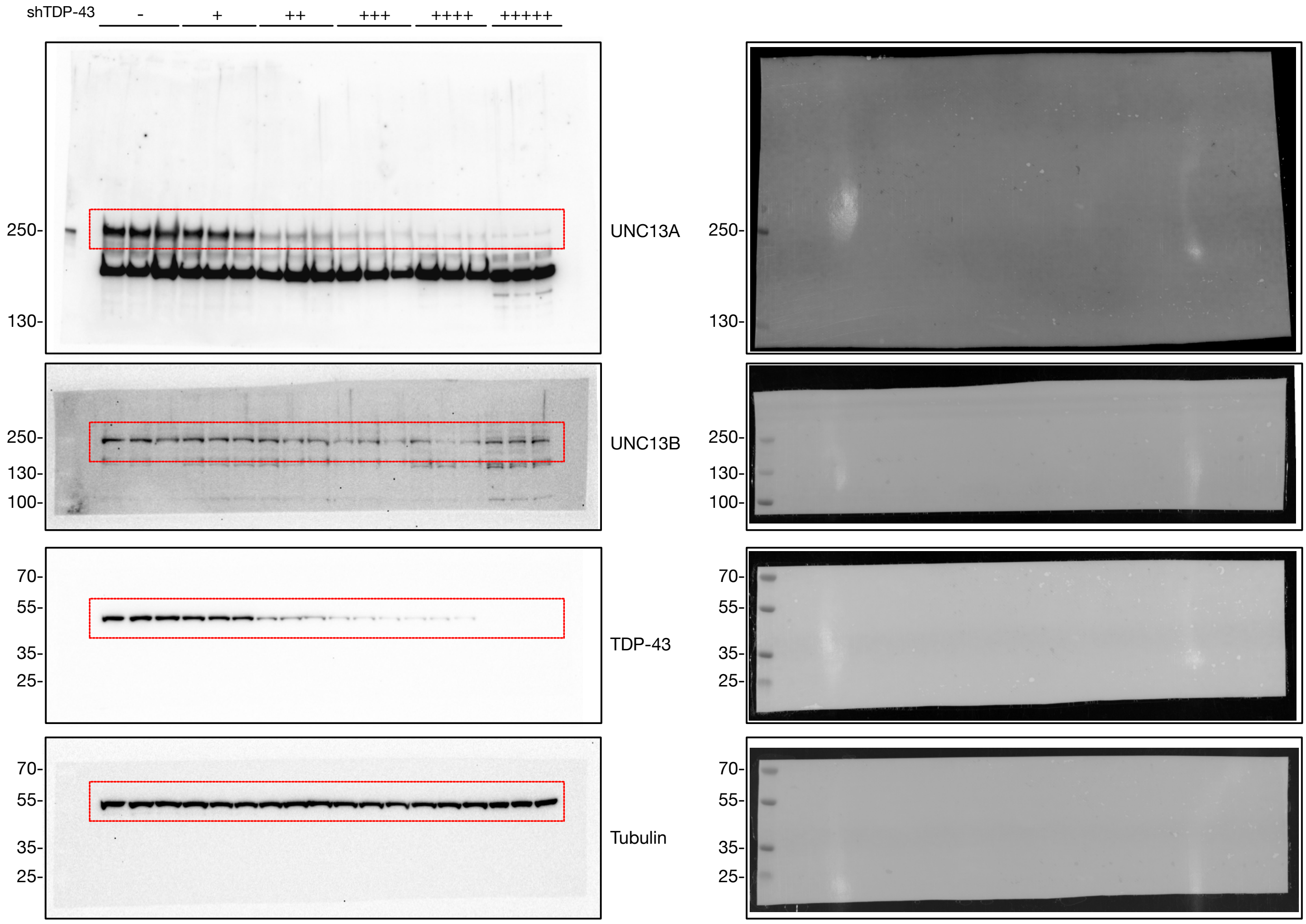

Supplement: Supplementary file 1 — Uncropped immunoblots from Extended Data Fig. 3f. Red dashed boxes indicate regions shown in the figure. UNC13B, Tubulin and TDP-43 are from the same membrane. UNC13A is blotted on a separate membrane. [file 41586_2022_4436_MOESM1_ESM.pdf]
