# Supplementary material for: TDP-43 loss and ALS-risk SNPs drive mis-splicing and depletion of UNC13A
Source: Nature. 2022 Feb 23;603(7899):131–7. doi: 10.1038/s41586-022-04436-3 (PMC8891020; doi:10.1038/s41586-022-04436-3)
Supplement: Supplementary file 2 — Reporting Summary [file 41586_2022_4436_MOESM2_ESM.pdf]

nature research

Corresponding author(s): Michael E. Ward, Pietro Fratta

Last updated by author(s): Dec 10, 2021

Reporting Summary

Nature Research wishes to improve the reproducibility of the work that we publish. This form provides structure for consistency and transparency in reporting. For further information on Nature Research policies, see our Editorial Policies and the Editorial Policy Checklist.

Statistics

For all statistical analyses, confirm that the following items are present in the figure legend, table legend, main text, or Methods section.

n/a	Confirmed	
☐	☒	The exact sample size (*n*) for each experimental group/condition, given as a discrete number and unit of measurement
☐	☒	A statement on whether measurements were taken from distinct samples or whether the same sample was measured repeatedly
☐	☒	The statistical test(s) used AND whether they are one- or two-sided<br>*Only common tests should be described solely by name; describe more complex techniques in the Methods section.*
☒	☐	A description of all covariates tested
☒	☐	A description of any assumptions or corrections, such as tests of normality and adjustment for multiple comparisons
☐	☒	A full description of the statistical parameters including central tendency (e.g. means) or other basic estimates (e.g. regression coefficient) AND variation (e.g. standard deviation) or associated estimates of uncertainty (e.g. confidence intervals)
☐	☒	For null hypothesis testing, the test statistic (e.g. *F*, *t*, *r*) with confidence intervals, effect sizes, degrees of freedom and *P* value noted<br>*Give P values as exact values whenever suitable.*
☒	☐	For Bayesian analysis, information on the choice of priors and Markov chain Monte Carlo settings
☒	☐	For hierarchical and complex designs, identification of the appropriate level for tests and full reporting of outcomes
☒	☐	Estimates of effect sizes (e.g. Cohen's *d*, Pearson's *r*), indicating how they were calculated

Our web collection on statistics for biologists contains articles on many of the points above.

Software and code

Policy information about availability of computer code

Data collection	Xcalibur v4.3
Data analysis	https://github.com/ulelab/ultraplex<br>https://github.com/frattalab/unc13a_cryptic_splicing<br>https://github.com/frattalab/rna_seq_snakemake<br>https://github.com/frattalab/splicing<br>https://github.com/frattalab/bedops_parse_star_junctions<br>https://github.com/frattalab/pipeline_iclip<br>MicroCal PEAQ-ITC<br>BWA-MEM v0.7.15<br>Genome Analysis Toolkit v3.5<br>LeafCutter - GitHub branch - 'psi_2019'<br>MAJIQ v2.1<br>Integrative Genomics Viewer v 2.9.1<br>Salmon v1.5.1<br>iCount v2.0.1.dev<br>STAR v2.7.0f & 2.7.2a<br>fastp v0.19.11<br>DESeq2 v1.30.1<br>Snakemake v5.5.4<br>IRFinder v1.3.0<br>bedtools v2.29.2<br>R v4.0.3

Proteome Discoverer (v2.4)
featureCounts v1.6.4
ultraplex v1.1.2
Bowtie2 v2.4.2
UMI-tools v1.0.1
LocusZoom
regtools v0.5.1
samtools v1.9
Picard Tools v2.4.1
ImageJ v1.52p

For manuscripts utilizing custom algorithms or software that are central to the research but not yet described in published literature, software must be made available to editors and reviewers. We strongly encourage code deposition in a community repository (e.g. GitHub). See the Nature Research guidelines for submitting code & software for further information.

Data

Policy information about availability of data

All manuscripts must include a data availability statement. This statement should provide the following information, where applicable:

- Accession codes, unique identifiers, or web links for publicly available datasets
- A list of figures that have associated raw data
- A description of any restrictions on data availability

Minimum data to reproduce results freely available: https://github.com/frattalab/unc13a_cryptic_splicing/tree/main/data
RNA-Seq Data for i3Neurons, SH-SY5Y and SK-N-DZa are available through the European Nucleotide Archive (ENA) under accession PRJEB42763(Fig 1,2, fig S1).
Public data was obtained from Gene Expression Omnibus (GEO): iPSC MNs (Klim et al., 2019)-GSE121569, SK-N-DZb-GSE97262, and FACS-sorted frontal cortex neuronal nuclei-GSE126543(Fig 1,2,3, fig S1).
Riboseq: E-MTAB-10235(Fig 2, fig S2).
Targeted RNA seq: E-MTAB-10237(Fig 4)
Minigene iCLIP: E-MTAB-10297(Fig 4)
NYGC ALS Consortium RNA-seq: RNA-Seq data generated through the NYGC ALS Consortium in this study can be accessed via the NCBI's GEO database (GEO GSE137810, GSE124439, GSE116622, and GSE153960). All RNA-Seq data generated by the NYGC ALS Consortium are made immediately available to all members of the Consortium and with other consortia with whom we have a reciprocal sharing arrangement. To request immediate access to new and ongoing data generated by the NYGC ALS Consortium and for samples provided through the Target ALS Postmortem Core, complete a genetic data request form at ALSData@nygenome.org.(Fig 3,4, fig S5,6,S8-11,S13)
NYGC ALS Consortium Whole Genome Seq: to be released later with companion manuscript.
NYGC ALS Consortium genotypes on common SNPs in this study rs129731921 and rs12608932 are present at https://github.com/frattalab/unc13a_cryptic_splicing/blob/main/data/nygc_junction_information.csv

Field-specific reporting

Please select the one below that is the best fit for your research. If you are not sure, read the appropriate sections before making your selection.

☒ Life sciences ☐ Behavioural & social sciences ☐ Ecological, evolutionary & environmental sciences

For a reference copy of the document with all sections, see nature.com/documents/nr-reporting-summary-flat.pdf

Life sciences study design

All studies must disclose on these points even when the disclosure is negative.

Sample size	Sample size in NYGC ALS Consortium was not determined in advance, the consortium data collection is ongoing, and sample size was determined by the number of available RNA-seq and genomically matched samples available at start time of analysis (2020-07). Sample size for RNA-seq on cell lines new to this study was determined by prior literature using similar experimental approaches rather than by power analysis. Examples of similar RNA-seq studies using similar (n of at least 3 in each condition) samples size include "Major hnRNP proteins act as general TDP-43 functional modifiers both in Drosophila and human neuronal cells" and "Premature polyadenylation-mediated loss of stathmin-2 is a hallmark of TDP-43-dependent neurodegeneration"
Data exclusions	Tissue samples with discordant genotype on rs12608932 and rs12973192 (2) were excluded from the analysis on genotype effect on the ratio of UNC13A/STMN2 cryptic PSI.
Replication	Expression of the UNC13A CE was replicated in multiple independent cell lines, WTC11 iPS and NCRM5 iPS cell lines. Minigene experiments were replicated in 3 biological replicates. SH-SY5Y doxycycline knock-downs were replicated in 3 biological replicates.
Randomization	Not relevant to this study as no randomization is required due to the homogeneous nature of the cell lines. Furthermore, our RNA-seq discovery assays are high-throughput and were initially done in a hypotheses-free analysis. Observations in the RNA-seq were validated and confirmed by biochemical assays to bolster initial observations from the high-throughput assays.
Blinding	BaseScope cortical sections were imaged and analysed blinded to disease status.

Reporting for specific materials, systems and methods

We require information from authors about some types of materials, experimental systems and methods used in many studies. Here, indicate whether each material, system or method listed is relevant to your study. If you are not sure if a list item applies to your research, read the appropriate section before selecting a response.

Materials & experimental systems

n/a	Involved in the study
☐	☒ Antibodies
☐	☒ Eukaryotic cell lines
☒	☐ Palaeontology and archaeology
☒	☐ Animals and other organisms
☐	☒ Human research participants
☒	☐ Clinical data
☒	☐ Dual use research of concern

Methods

n/a	Involved in the study
☒	☐ ChIP-seq
☒	☐ Flow cytometry
☒	☐ MRI-based neuroimaging

Antibodies

Antibodies used

Rabbit anti-UNC13A (Synaptic Systems 126 103) dilution 1:2,000
Rabbit anti-UNC13B (abcam ab97924) dilution 1:1,000
Rat anti-Tubulin (abcam ab6161 clone YOL1/34) dilution 1:5,000
Mouse anti-TDP-43 (abcam ab104223 clone 3H8) dilution 1:5,000
Rabbit anti-TDP-43 10782-2-AP (Proteintech) - lot 00065465
Goat anti-Rabbit HRP (Bio-Rad 1706515) 1:10,000
Goat anti-Mouse HRP (Bio-Rad 1706516) 1:10,000
Rabbit anti-Rat HRP (Dako P0450) 1:10,000
Donkey anti-Rabbit 488 (Jackon Immuno 711-545-152)
Donkey anti-Mouse 647 (Jackson Immuno 715-605-151)
Rabbit anti-TDP-43 (Proteintech 12892-1-AP)
Mouse anti-TUBB3 (Biolegend 801201)

Validation

Mouse anti-TDP43 abcam ab104223 was validated via shRNA experiments against TDP-43

Rb anti-UNC13A (synaptic systems 126 103) has been previously verified in KO mouse [Lai Y, Choi UB, Leitz J, Rhee HJ, Lee C, Altas B, Zhao M, Pfuetzner RA, Wang AL, Brose N, Rhee J, et al. Molecular Mechanisms of Synaptic Vesicle Priming by Munc13 and Munc18. Neuron (2017) 953: 591-607.e10.]. The antibody was additionally validated by western blot analysis of human brain lysate and U2OS cell lysate which do not express UNC13A. As well as overexpression experiments with GFP tagged UNC13A.

Rb anti-UNC13B (abcam ab97924) was validated with overexpression experiments with GFP tagged UNC13B.

Rat anti-Tubulin (abcam ab6161) has been cited in > 126 publications.
Rabbit anti-TDP-43 10782-2-AP (Proteintech) has been cited in >1200 publications. Rabbit anti-TDP-43 10782-2-AP (Proteintech) has postive WB detection in SH-SY5Y cells, HeLa cells, K-562 cells, C2C12 cells, Neuro-2a cells, Positive IHC detected in mouse brain tissue, human brain tissue, human brain (FTLD-U) tissue, human gliomas tissue, human pancreas tissue, rat brain tissue. Positive IF detected in HeLa cells, SH-SY5Y cells,Positive FC detected in HeLa cells.

Goat anti-Rabbit HRP (Bio-Rad 1706515) according to manufacturer's website has been double-affinity purified with human IgG adsorbed.
Goat anti-Mouse HRP (Bio-Rad 1706516) has been used in >1000 citations.
Rabbit anti-Rat HRP (Dako P0450) has been used in 120 citations.
Donkey anti-Rabbit 488 (Jackon Immuno 711-545-152) has been used in 705 citations - https://www.jacksonimmuno.com/catalog/products/711-545-152
Donkey anti-Mouse 647 (Jackson Immuno 715-605-151) has been used in 121 citations - https://www.jacksonimmuno.com/catalog/products/715-605-151
Rabbit anti-TDP-43 (Proteintech 12892-1-AP) has been used in 321 citations https://www.ptglab.com/Products/TARDBP-Antibody-12892-1-AP.htm
Mouse anti-TUBB3 (Biolegend 801201)has been used in 597 citations https://www.biolegend.com/en-us/search-results/purified-anti-tubulin-beta-3-tubb3-antibody-11580

Eukaryotic cell lines

Policy information about cell lines

Cell line source(s)

iPS-derived cortical neurons are from the WTC11 line, which was derived from a healthy human male participant and NCRM5, which was derived from a healthy human male's cord blood and obtained from the Coriell cell repository. All policies of the NIH Intramural Research Program for the registration and use of this iPS cell line were followed. HEK293T and

	SH-SY5Y cells were obtained from ATCC. SK-N-DZ cells were obtained from the International Centre for Genetic Engineering and Biotechnology in Trieste, Italy.
Authentication	WTC11 iPS cell line was validated to have a normal male karyotype<br>NCRM5 iPS cell line was validated to have a normal male karyotype
Mycoplasma contamination	WTC11 iPS cell line was confirmed to be mycoplasma free based on the Lonza MycoAlert mycoplasma testing kit.<br>NCRM5 iPS cell line was confirmed to be mycoplasma free based on the Lonza MycoAlert mycoplasma testing kit.
Commonly misidentified lines<br>(See ICLAC register)	No commonly misidentified cell lines were used in this study.

Human research participants

Policy information about studies involving human research participants

Population characteristics	1349 tissue samples from 377 unique participants (164 female). 77 controls - Median age 67, 239 ALS - Median age 66, 61 FTLD - Median age 67
Recruitment	In NYGC ALS Consortium recruitment and contribution postmortem samples and clinical information was performed by members using their recruitment criteria and strategy.
Ethics oversight	The NYGC ALS Consortium samples presented in this work were acquired through various IRB protocols from member sites and the Target ALS postmortem tissue core and transferred to the NYGC in accordance with all applicable foreign, domestic, federal, state, and local laws and regulations for processing, sequencing, and analyses. The Biomedical Research<br>3<br>Alliance of New York (BRANY) IRB serves as the central ethics oversight body for NYGC ALS Consortium. Ethical approval was given and is effective through 08/22/2022.

Note that full information on the approval of the study protocol must also be provided in the manuscript.
